# A multicenter performance evaluation of the new Elecsys Vitamin D total III assay versus reference isotope dilution liquid chromatography tandem mass spectrometry and commercially available comparators

**DOI:** 10.1002/jcla.24610

**Published:** 2022-07-19

**Authors:** Peter Findeisen, Marta Leis, Garnet Bendig, Jill Grimme, Elizabeth Moser, Christian Vogl, Robert Christenson

**Affiliations:** ^1^ MVZ Labor Dr. Limbach & Kollegen Heidelberg Germany; ^2^ TRIGA‐S Habach Germany; ^3^ Roche Diagnostics GmbH Penzberg Germany; ^4^ Roche Diagnostics Indianapolis Indiana USA; ^5^ University of Maryland School of Medicine Baltimore Maryland USA

**Keywords:** analytical performance, assay, method comparison, vitamin D

## Abstract

**Background:**

Vitamin D deficiency/insufficiency and toxicity are worldwide issues; thus, accurate diagnostic assays are required to measure vitamin D. We evaluated the performance of the new Elecsys^®^ Vitamin D total III assay (Roche Diagnostics International Ltd).

**Methods:**

Repeatability and intermediate precision of the Elecsys Vitamin D total III assay (cobas e 601 analyzer) were evaluated at three sites using five human serum pools (HSPs) and two PreciControls (five‐day model, one reagent lot [CLSI‐EP05‐A3]) and compared against prespecified acceptance criteria. A serum verification panel, with reference isotope dilution liquid chromatography tandem mass spectrometry (ID‐LC–MS/MS) values, was used for comparator assay/concordance studies at two sites, assessed using unweighted Deming regression. Testing of serum vs. plasma on the Elecsys assay was conducted at one site using samples from healthy adults; assessed using Passing‐Bablok regression.

**Results:**

Repeatability (HSP1 [16.8–18.4 ng/ml], SD 0.87–1.07; HSP5 [94.5–98.0 ng/ml], CV 1.58%–2.76%) and intermediate precision (HSP1, SD 1.14–1.77; HSP5, CV 2.00%–4.13%) met acceptance criteria across sites. Agreement was observed between the Elecsys assay and (i) the ID‐LC–MS/MS verification panel (slope, 0.936–1.01; Pearson's r, 0.960–0.986) and (ii) comparator assays (slope, 0.921–1.15; Pearson's r, 0.958–0.982). The Elecsys assay correctly assigned the highest combined percentage of samples to deficient (100%) and insufficient (89.5%) vitamin D categories vs. comparator assays and demonstrated comparable performance in serum and plasma (y = 0.103 + 0.984x).

**Conclusions:**

The Elecsys Vitamin D total III assay demonstrated good analytical performance and compared favorably with other assays, supporting its use in clinical practice.

## Graphical abstract

This was a multicenter performance evaluation of the new Elecsys^®^ Vitamin D total III assay (Roche Diagnostics International Ltd), intended for the quantitative determination of total 25‐hydroxyvitamin D in adult human serum and plasma. The Elecsys Vitamin D total III assay demonstrated good analytical performance and compared favorably with other assays, supporting its use in routine laboratory practice.

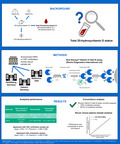

## 1 INTRODUCTION

Vitamin D is an essential nutrient for humans; it is primarily obtained through exposure to ultraviolet B (UVB) radiation in sunlight and dietary intake. Naturally occurring dietary sources rich in vitamin D are limited, and therefore, the majority of vitamin D obtained through diet is from fortified foods and supplements.^1^ Alongside its critical role in calcium homeostasis and bone mineralization, vitamin D has roles in fundamental cellular functions such as cell growth, immune function, and glucose metabolism, amongst others.^2^, ^3^, ^4^

Vitamin D exists in two bioequivalent forms, vitamins D_2_ and D_3_, which are readily absorbed in the small intestine and converted to 25‐hydroxyvitamin D in the liver. Vitamin D_2_ mainly comes from plant sources and fortified foods, whereas vitamin D_3_ is produced in the skin by exposure to UVB or digested from animal‐sourced foods. In the kidney, 25‐hydroxyvitamin D is converted into the physiologically active form, 1,25‐dihydroxyvitamin D.^2^ 25‐hydroxyvitamin D has a circulating half‐life of 15 days,^2^ whereas the half‐life of circulating 1,25‐dihydroxyvitamin D is 4–6 h.^5^ Furthermore, the concentration of 1,25‐dihydroxyvitamin D in serum is 1000‐fold less than 25‐hydroxyvitamin D.^5^ The serum concentration of total 25‐hydroxyvitamin D (sum of 25‐hydroxyvitamin D_2_ and 25‐hydroxyvitamin D_3_) is the most reliable indicator of vitamin D status.^5^

It is estimated that 1 billion people have vitamin D deficiency or insufficiency worldwide.^6^ Controversy surrounds the definitive concentration of 25‐hydroxyvitamin D in serum that is indicative of vitamin D deficiency^7^; however, expert bodies have stated that a serum concentration of 30 ng/ml is necessary to maximize the effect of vitamin D on overall health.^8^ In contrast, the widespread increased intake of supplements with a higher than recommended daily allowance of vitamin D can be associated with exogenous hypervitaminosis D and hypercalcemia also known as vitamin D toxicity; however, this is very rare.^9^

Due to the increasing number of publications on the role of vitamin D in other disease areas^6^ beyond its well‐documented role in bone health,^10^ the number of requests by healthcare providers for total 25‐hydroxyvitamin D tests has increased, making testing on automated instruments part of standard routine measurements. However, routine screening for total 25‐hydroxyvitamin D is not currently recommended by clinical societies.^11^, ^12^ Dependent upon severity, people who present with vitamin D deficiency or insufficiency can be treated with lifestyle changes or pharmaceutical intervention using over‐the‐counter or high‐dose vitamin D supplements.^13^ Reliable diagnostic assays designed to measure total 25‐hydroxyvitamin D are required to identify individuals with vitamin D insufficiency/deficiency and inform treatment. In addition, there is no vitamin D_3_ supplement currently approved by the US Food and Drug Administration; thus, the dose of vitamin D_3_ in some supplements may be inconsistent and the use of a regulated total vitamin D diagnostic assay is key to determining the vitamin D status of individuals using these supplements.

The gold standard for the measurement of total 25‐hydroxyvitamin D in serum is isotope dilution liquid chromatography tandem mass spectrometry (ID‐LC–MS/MS); however, most routine analyses for total 25‐hydroxyvitamin D are performed by automated assays.^14^, ^15^ In the past, a range of commercially available assays have shown a general lack of analytical precision at the extremes of total 25‐hydroxyvitamin D concentration, as well as substantial variation in measurement between platforms^16^; therefore, the International Vitamin D Standardization‐Certification Program was recently set up by the Centers for Disease Control and Prevention (CDC) with the aim of standardizing the measurement of total 25‐hydroxyvitamin D in serum using a higher‐order reference measurement procedure.^17^

The new Elecsys^®^ Vitamin D total III assay (Roche Diagnostics International Ltd, Rotkre) is intended for the quantitative determination of total 25‐hydroxyvitamin D in serum and plasma in adults. This method has been standardized using internal calibrators that are traceable to the ID‐LC–MS/MS 25‐hydroxyvitamin D Reference Measurement Procedure.^18^, ^19^ The ID‐LC–MS/MS is traceable to the National Institute of Standards and Technology Standard Reference Material 2972.^20^

The aims of this study were to evaluate the analytical performance of the new Elecsys Vitamin D total III assay, to calculate the accuracy of the Elecsys assay vs. reference ID‐LC–MS/MS values, to conduct method comparison vs. other commercially available assays, and to test serum vs. plasma samples on the cobas e 601 analyzer.

## 2 MATERIALS AND METHODS

### 2.1 Study design

This was a multicenter study conducted from February to March 2020 at two sites in Germany (MVZ Labor Dr. Limbach & Kollegen, Heidelberg and TRIGA‐S, Habach) and one site in the United States (University of Maryland School of Medicine, Baltimore). Precision and method comparison experiments were performed under routine conditions. All sites were equipped with a cobas e 601 analyzer (Roche Diagnostics International Ltd).

Prior to commencement of the main study, each site was required to complete an initial familiarization phase consisting of a repeatability (within‐run precision) test. To monitor the consistent quality of the results, each site was required to run a daily quality control prior to each experimental run and to proceed only if within the target ranges of the assay. The medical decision point (MDP) for the concentration of total 25‐hydroxyvitamin D in serum was defined as 30 ng/ml.

### 2.2 Samples and sample handling

For the precision experiment, anonymized human serum pools (HSPs) without clinical or demographic information were supplied by Roche Diagnostics. For method comparison vs. other commercially available assays and concordance analyses, a CDC verification human serum sample set (CDC; *n* = 120) derived from single donors with predefined reference target values of vitamin D (14.1–383 nmol/L [4.1–110.5 ng/ml]) was used.^15^ Of note, 36 of the 120 samples contained both 25‐hydroxyvitamin D_2_ and 25‐hydroxyvitamin D_3_ based on the ID‐LC–MS/MS values for the respective D_2_ and D_3_ forms reported, in addition to the total target values. The target values of the CDC verification set were determined using ID‐LC–MS/MS and are used for the purposes of verifying the standardization of methods quantifying total 25‐hydroxyvitamin D.

For the comparison of the Elecsys Vitamin D total III assay performance in serum vs. plasma, paired serum and plasma (lithium‐heparin) samples from 462 apparently healthy individuals from three geographically diverse locations across the United States (Prism Research; Vanderbilt University School of Medicine; and Century Clinical Family Research, representing Northern, Mid and Southern regions, respectively) collected during the summer and winter seasons were measured. Individuals could only donate a sample once (i.e., a participant who had contributed a specimen to the summer campaign could not contribute during the winter campaign). Participants were balanced with respect to sex and a minimum of 30% of participants were dark‐skinned or light‐skinned, respectively.

All samples were shipped and stored as required at 2–8°C, −20°C, or −80°C until testing.

### 2.3 Ethics statement

This study was conducted in accordance with the principles of the Declaration of Helsinki. For the precision and method comparison experiments, ethics approval was not required for the two German centers in accordance with local legislation. Institutional review board (IRB) approval was granted by the University of Maryland in December 2019 (IRB reference number HP‐00089042). For the testing of serum vs. plasma, each of the three collection sites received IRB approval in June 2019 (IRB reference numbers 191101, 20191597, and 00003657); IRB approval was granted to the testing site (Washington University) in December 2019 (IRB reference number 201912096).

### 2.4 Elecsys Vitamin D total III assay

**FIGURE 1:**
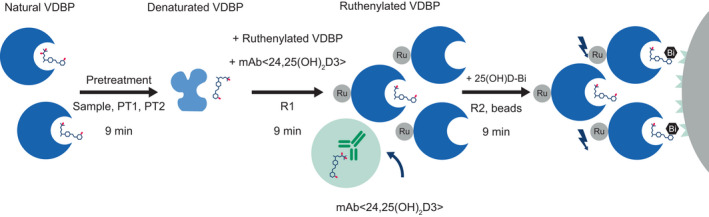
Assay principle of the Elecsys Vitamin D total III assay. PT1, pretreatment 1; PT2, pretreatment 2; R1, reagent 1; R2, reagent 2; VDBP, vitamin D binding protein.

The Elecsys Vitamin D total III assay is an electrochemiluminescence binding assay based on the principle of back‐titration (Figure 1). The new generation assay was developed to improve the general performance as well as mitigate biotin interference with the assay, relative to previous generations. During pretreatment, bound 25‐hydroxyvitamin D is released from vitamin D‐binding protein (VDBP) in the sample. During the second incubation, the pre‐treated sample is mixed with the ruthenium‐labelled VDBP and a complex between the total 25‐hydroxyvitamin D and the ruthenylated VDBP is formed. A specific unlabeled antibody binds to 24,25‐dihydroxyvitamin D present in the sample to inhibit cross‐reactivity with this vitamin D metabolite. In the final step, streptavidin‐coated microparticles and biotin‐labelled 25‐hydroxyvitamin D are added and unbound ruthenylated VDBP become occupied. A complex comprising the ruthenylated VDBP and the biotinylated 25‐hydroxyvitamin D is formed and becomes bound to the solid phase via interaction of biotin and streptavidin.

### 2.5 Precision experiments

A five‐day precision experiment was conducted across all three sites. Repeatability (within‐run precision) and intermediate (within‐lab) precision for the Elecsys Vitamin D total III assay were measured in five HSPs (Roche Diagnostics) and two PreciControls (PC1 and PC2; Roche Diagnostics), using one reagent lot per site per CLSI‐EP05‐A3 guidance. Standard deviation (SD) and coefficient of variation (CV) values were calculated and compared against prespecified acceptance criteria.

### 2.6 Method comparison vs. commercially available comparator assays

Method comparison of the Elecsys Vitamin D total III assay against four commercially available comparator assays was conducted at two of the three sites: Heidelberg, Germany (ADVIA Centaur Vitamin D Total; ARCHITECT 25‐OH Vitamin D; LIAISON 25 OH Vitamin D TOTAL) and Baltimore, MD, USA (Access 25 [OH] Vitamin D Total). One of the 120 samples in the CDC verification serum sample set was outside the measuring range of the Access 25 (OH) Vitamin D Total assay, while an additional three samples were outside the measuring range of all four assays (Table 1); therefore, all were excluded from the analysis.

**TABLE 1:** Vitamin D measuring ranges for the Elecsys Vitamin D total III assay and comparator assays

Assay^a^	Manufacturer	Vitamin D measuring range, ng/ml	Repeatability (within‐run precision)		Intermediate precision (within‐lab/total imprecision)	
			SD	CV, %	SD	CV, %
Elecsys Vitamin D total III^23^	Roche	3.0–120.0	0.9–3.4	2.3–7.4	1.2–3.7	3.3–9.8
Access 25 (OH) Vitamin D Total^29^	Beckman Coulter	7.0–120.0	0.5–1.6	1.5–3.8	1.0–7.5	6.8–7.7
ADVIA Centaur Vitamin D Total^30^	Siemens	4.2–150.0	0.6–3.4	3.0–5.3	1.6–4.7	4.2–11.9
ARCHITECT 25‐OH Vitamin D^31^	Abbott	3.4–155.9	0.3–6.2	1.8–5.1	0.4–7.3	2.3–7.1
LIAISON 25 OH Vitamin D TOTAL^32^	DiaSorin	4.0–150.0	1.7–2.0	4.9–5.4	3.8–4.1	7.8–10.6

Abbreviations: CV, coefficient of variation; ID‐LC–MS/MS, isotope dilution liquid chromatography tandem mass spectrometry; SD, standard deviation.

^a^ Vitamin D measuring range, repeatability, and intermediate precision taken from the respective package inserts for each assay. All assays were standardized using standards traceable to the ID‐LC–MS/MS 25‐hydroxyvitamin D Reference Measurement Procedure.^14^, ^15^

Quantitative comparison was conducted using λ values specific to each comparator assay. For each comparator, total intermediate precision SDs (x) and their corresponding mean concentration levels (y) (within the measurement range of the Elecsys Vitamin D total III assay), as provided in the respective method sheets, were used to derive the slope (b) and intercept (m) values utilizing the following equations:

$\text{Slope Equation}:b=\sum \left(x-\overset{-}{x}\right)\;\left(y-\overset{-}{y}\right)/\sum {\left(x-\overset{-}{x}\right)}^{2}$

$\text{Intercept Equation}:m=\overset{-}{y}-b\overset{-}{x}$

$\mathrm{SD}\;\mathrm{at}\;30\;\mathrm{ng}/\mathrm{ml}:\mathrm{SD}=\left(m\times 30\right)+b$

These values were then used to estimate each comparator's SD at the MDP of 30 ng/ml, which was used to calculate a comparison‐specific λ value for use in unweighted Deming regression.

### 2.7 Concordance analysis

Concordance between the Elecsys Vitamin D total III, ADVIA Centaur Vitamin D Total, ARCHITECT 25‐OH Vitamin D, and LIAISON 25 OH Vitamin D TOTAL assays and the target values of the CDC verification sample set were analyzed at one site (Heidelberg, Germany). Samples were classified as deficient, insufficient, or sufficient according to their measurement value for each assay, as was their CDC measurement. The rate of agreement between the assay‐specific and CDC classifications was then determined. In addition, concordance between the Access 25 (OH) Vitamin D Total assay and target values of the CDC verification sample set was analyzed at one site (Baltimore, MD, USA). The concordance in sample classification for each of the following vitamin D status groups was calculated for each assay: deficient (<20 ng/ml), insufficient (20–30 ng/ml), and sufficient (>30 ng/ml).

### 2.8 Serum versus plasma sample analysis

Measurement of 25‐hydroxyvitamin D using the Elecsys Vitamin D total III assay in serum and plasma was conducted at an independent site (Washington University). Between‐matrix differences were assessed using Passing–Bablok regression and Bland–Altman analyses.

### 2.9 Data management and analyses

The Elecsys Vitamin D total III assay output was directly captured by Windows‐based computer‐aided evaluation (WinCAEv) software (Roche Diagnostics GmbH). SAS version 9.4 (SAS Institute) and/or R version 3.5.1 (The R Foundation) and BioWarp (Roche Diagnostics GmbH) were used for data analyses.

For the method comparison, outliers were detected using a modified version of the Grubbs‐test, defined by the median and MD68 statistic. In brief, the “normal” variability of a given sample was calculated using the MD68 statistic. For every sample/site, the median of five measurements per day was calculated, as well as the difference between each measurement within the day‐to‐day median. If the absolute difference of a particular data point was greater than 2.75 × MD68, this data point was detected as an outlier. Exclusion of one outlying data point was permitted per the study protocol.

## 3 RESULTS

The Elecsys Vitamin D total III assay demonstrated low SD and CV values when measuring 25‐hydroxyvitamin D in HSPs and two PreciControl samples (Table 2). For the lowest concentration sample (HSP1, 16.8–18.4 ng/ml), repeatability and intermediate precision ranged between SD 0.87–1.07 and SD 1.14–1.77, respectively, across the three study sites. For the highest concentration sample (HSP5, 94.5–98.0 ng/ml), repeatability and intermediate precision ranged between CV 1.58%–2.76% and CV 2.00%–4.13%, respectively, across the three study sites. There were three outliers identified using predefined criteria: HSP2 (Heidelberg), HSP3 (Habach), and HSP4 (Habach). For two of the outlying data points (HSP2, Heidelberg; HSP3, Habach), analysis showed that their inclusion did not affect the ability of the assay to meet the acceptance criteria. The third data point was excluded according to the standard operating procedure, and the remaining dataset met the acceptance criteria. At the Baltimore site, all results met the acceptance criteria.

**TABLE 2:** Precision of the Elecsys Vitamin D total III assay across the three study sites

Specimen	Site	Sample number, *n*	Mean vitamin D concentration, ng/ml	Repeatability (within‐run precision)		Intermediate precision (within‐lab/total imprecision)	
				SD	CV, %	SD	CV, %
HSP1	Heidelberg	25	17.8	0.94	5.27	1.24	6.98
	Habach	25	18.4	1.07	5.81	1.77	9.58
	Baltimore	25	16.8	0.87	5.17	1.14	6.78
HSP2	Heidelberg^a^	24	34.0	0.79	2.33	1.10	3.22
	Habach	25	34.6	1.79	5.19	2.71	7.83
	Baltimore	25	32.1	1.04	3.23	1.43	4.46
HSP3	Heidelberg	25	63.7	1.76	2.76	2.02	3.16
	Habach^a^	24	64.3	3.84	5.97	5.38	8.37
	Baltimore	25	61.7	1.80	2.92	1.97	3.20
HSP4	Heidelberg	25	82.8	2.76	3.33	2.76	3.33
	Habach^b^	24	82.1	5.28	6.43	6.29	7.66
	Baltimore	25	80.2	2.01	2.51	2.48	3.10
HSP5	Heidelberg	25	96.4	1.75	1.82	1.93	2.00
	Habach	25	98.0	2.70	2.76	4.05	4.13
	Baltimore	25	94.5	1.49	1.58	2.41	2.55
PC1	Heidelberg	25	20.9	0.82	3.91	1.12	5.35
	Habach	25	21.1	1.29	6.13	2.04	9.71
	Baltimore	25	19.8	0.88	4.42	1.05	5.29
PC2	Heidelberg	25	39.4	1.05	2.68	1.25	3.18
	Habach	25	40.1	2.25	5.61	2.75	6.87
	Baltimore	25	38.3	0.96	2.50	1.37	3.58

Abbreviations: CV, coefficient of variation; HSP, human serum pool; PC, PreciControl; SD, standard deviation.

^a^ One outlier was identified; however, analysis showed that the acceptance criteria had been fulfilled irrespective of whether this outlier was or was not included.

^b^ The acceptance criteria for repeatability were not fulfilled with the outlier included. This outlier was excluded based upon the standard operating procedure.

**FIGURE 2:**
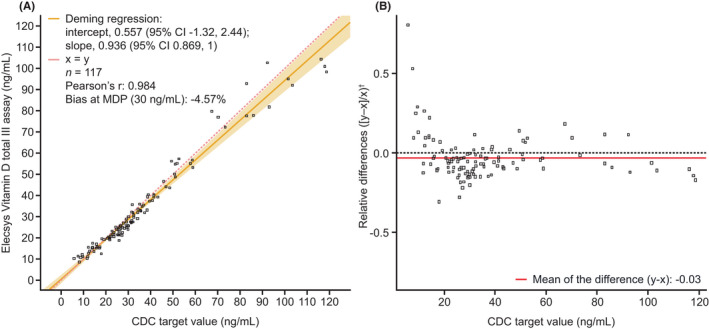
Comparison of the Elecsys Vitamin D total III assay versus CDC target values at Heidelberg in (A) an Unweighted Deming regression fit plot and (B) a Bland–Altman plot. ^†^Relative differences were calculated using standardized values to allow comparison across the entire measuring range. CDC, Centers for Disease Control and Prevention; CI, confidence interval; MDP, medical decision point.

The Elecsys Vitamin D total III assay showed excellent agreement with the target concentration of total 25‐hydroxyvitamin D measured by ID‐LC–MS/MS in the CDC verification serum sample set (Figure 2A). The Deming regression slope ranged from 0.936 to 1.01 across sites, Pearson's r ranged from 0.960 to 0.986, and bias at the MDP (30 ng/ml) ranged between −8.11% and 12.7%. The mean relative difference in vitamin D levels measured by the Elecsys Vitamin D total III assay vs. CDC target values was −0.03% or −3% (Figure 2B). All samples met the prespecified acceptance criteria.

**FIGURE 3:**
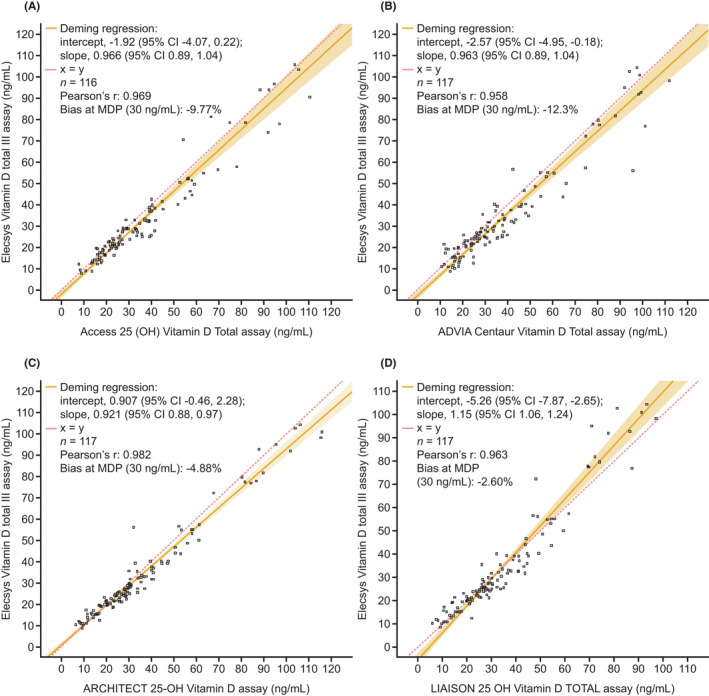
Method comparison of the Elecsys Vitamin D total III assay vs. (A) Access 25 (OH) Vitamin D Total, (B) ADVIA Centaur Vitamin D Total, (C) ARCHITECT 25‐OH Vitamin D, and (D) LIAISON 25 OH Vitamin D TOTAL assays. CI, confidence interval; MDP, medical decision point.

Using the CDC ID‐LC–MS/MS verification serum sample set, the Elecsys Vitamin D total III assay showed good agreement with the comparator assays (Access 25 [OH] Vitamin D Total, ADVIA Centaur Vitamin D Total, ARCHITECT 25‐OH Vitamin D, and LIAISON 25 OH Vitamin D TOTAL; Figure 3; Table S1).

Of the methods examined, the Elecsys Vitamin D total III assay demonstrated the best overall concordance with the CDC ID‐LC–MS/MS verification sample set, that is, it correctly identified the highest combined percentage of samples as deficient (100%) and insufficient (89.5%) in vitamin D versus comparator assays (Table 3). This was followed by the ARCHITECT 25‐OH Vitamin D assay, which correctly identified 100% of samples as deficient and 86.8% as insufficient in vitamin D. The Elecsys Vitamin D total III assay identified 85.5% of samples sufficient in vitamin D, while the Access 25 (OH) Vitamin D Total, ADVIA Centaur Vitamin D Total, ARCHITECT 25‐OH Vitamin D, and LIAISON 25 OH Vitamin D TOTAL classified 83.3%, 92.7%, 94.5%, and 90.9%, respectively.

**TABLE 3:** Concordance of the Elecsys Vitamin D total III assay vs. commercially available comparators in the CDC verification serum sample set

CDC group (*n*)	Vitamin D concentration, ng/ml	Concordant samples, *n* (%)				
		Elecsys Vitamin D total III	Access 25 (OH) Vitamin D Total	ADVIA Centaur Vitamin D Total	ARCHITECT 25‐OH Vitamin D	LIAISON 25 OH Vitamin D total
Deficient (24)	<20	24 (100.0)	23 (95.8)	21 (87.5)	24 (100.0)	23 (95.8)
Insufficient (38)	20–30	34 (89.5)	23 (60.5)	24 (63.2)	33 (86.8)	29 (76.3)
Sufficient (55)	>30	47 (85.5)	45 (83.3)^a^	51 (92.7)	52 (94.5)	50 (90.9)
Total (117)		105 (89.7)	91 (78.4)^a^	96 (82.1)	109 (93.2)	102 (87.2)

Abbreviation: CDC, Centers for Disease Control and Prevention.

^a^ For samples tested using the Access 25 (OH) Vitamin D Total assay (Beckman Coulter), one sample was outside the measuring range, as such, there were *n* = 54 samples in the sufficient group, and thus *N* = 116 samples in total.

**FIGURE 4:**
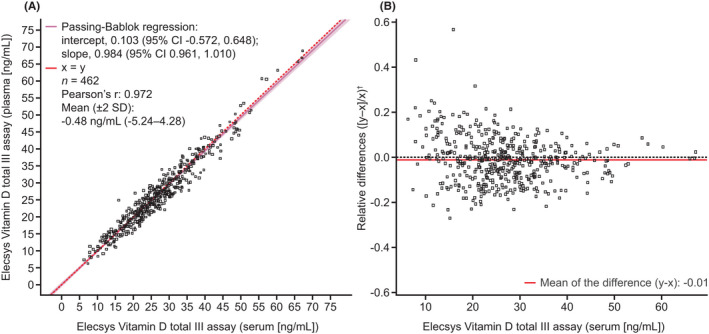
Comparison of the Elecsys Vitamin D total III assay in serum and plasma shown in (A) a Passing‐Bablok regression fit plot and (B) a Bland–Altman plot. ^†^Relative differences were calculated using standardized values to allow comparison across the entire measuring range. CI, confidence interval; SD, standard deviation.

The Elecsys Vitamin D total III assay demonstrated comparable analytical performance in serum and plasma samples from apparently healthy individuals (*n* = 462; Passing‐Bablok regression, y = 0.103 [95% confidence interval (CI), −0.572, 0.648] + 0.984x [95% CI, 0.961, 1.010]; mean [±2 SD]: ‐0.48 ng/ml [−5.24, 4.28]) (Figure 4A). The mean relative difference in vitamin D levels measured by the Elecsys Vitamin D total III assay in serum and plasma was −0.01% or −1% (Figure 4B).

## 4 DISCUSSION

In this multicenter study, the Elecsys Vitamin D total III assay showed good analytical performance across all three sites, and against the CDC ID‐LC–MS/MS verification sample set for the measurement of serum 25‐hydroxyvitamin D.^17^, ^21^ The Elecsys Vitamin D total III assay compared favorably with other commercially available comparator assays. The Elecsys assay correctly assigned the highest combined percentage of serum samples to deficient and insufficient vitamin D categories as outlined by the CDC Standardization‐Certification Program.^17^ The Elecsys Vitamin D total III assay results were equivalent in a large number of serum and plasma samples across a wide range of vitamin D concentrations. This study presents a comparative analysis of the new generation Elecsys Vitamin D total III assay for the measurement of total 25‐hydroxyvitamin D, which can be used as a reliable method for routine laboratory analysis.

There was similar agreement between the Elecsys Vitamin D total III assay and CDC ID‐LC–MS/MS reference sample set, as seen in an earlier study using the previous generation of the assay (Elecsys Vitamin D total II). The Elecsys Vitamin D total II assay has a similar formulation and the same standardization process as the Elecsys Vitamin D total III assay.^22^, ^23^ Broders et al. reported a Deming regression of y = 0.954x ‐ 0.707 and Pearson's r of 0.982 between the measurement of 25‐hydroxyvitamin D using the Elecsys Vitamin D total II and the CDC ID‐LC–MS/MS verification sample set.^24^ In addition, the investigators demonstrated the standardization to the ID‐LC–MS/MS method using samples from the College of American Pathologists (CAP) and vitamin D External Quality Assessment Scheme (DEQAS) with a mean recovery of 99% (83%–111%, CAP Accuracy Based Vitamin D samples 1–12) and 100% (84%–110%, DEQAS samples 476–490), respectively. The availability of an accurate, fully automated assay to measure 25‐hydroxyvitamin D offers advantages for clinical application over LC–MS/MS, such as increased automation, high throughput, and faster turnaround time.^16^

Some aspects of the methodology differ between the Elecsys Vitamin D total III assay and the comparator assays used in this study. First, the Elecsys Vitamin D total III assay is unique among the comparators tested as it uses recombinant VDBP for capturing 25‐hydroxyvitamin D_2_ and D_3_ rather than an antibody approach. Second, the Elecsys Vitamin D total III assay also utilizes an antibody for 24,25‐dihydroxyvitamin D to mitigate bias of vitamin D measurements caused by potential cross‐reactivity to this metabolite. Previous studies show that 24,25‐dihydroxyvitamin D may support the positive bias observed in other assays vs. LC–MS/MS‐derived measurements of 25‐hydroxyvitamin D, particularly in individuals with high vitamin D levels.^25^, ^26^ Finally, variability exists between assays due to the level of interference from other hydroxylated vitamin D metabolites, the hydrophobic nature of the molecule itself, the differential recognition of 25‐hydroxyvitamin D_2_ and 25‐hydroxyvitamin D_3,_ and the ability to separate 25‐hydroxyvitamin D from its serum binding partners.^27^

In this study, the Elecsys Vitamin D total III assay demonstrated good performance relative to comparator assays for identifying samples that were deficient and insufficient for 25‐hydroxyvitamin D. This is clinically advantageous when using the assay to identify individuals in need of supplementation as it permits accurate measurement of low levels of vitamin D and correct classification of patients. These findings provide support for the use of the Elecsys Vitamin D total III assay for the diagnosis of vitamin D deficiency and insufficiency in routine clinical practice.

The Elecsys Vitamin D total III assay showed comparable performance in serum and plasma samples. This suggests that the limitations of the previous generation assay (Elecsys Vitamin D total II) have been addressed and there is no longer a need for an additional sample preparation step^22^ or the use of cost‐prohibitive Barricor collection tubes for lithium‐heparin plasma^28^ when using the Elecsys Vitamin D total III assay.

The strengths of this study include the assessment of the assays across multiple sites in different countries, indicating reproducibility of the findings, and the measurement of a wide range of serum concentrations of total 25‐hydroxyvitamin D allowing bias at the MDP to be calculated. The assessment of multiple comparator assays in tandem also allowed for direct comparisons to be drawn between assays. Regarding limitations, while data on potential interference with hemolysis, bilirubin, and lipids are available, this was not assessed at external sites. In addition, as all samples were de‐identified, data on clinical characteristics and patient demographics were not available. Data are shown on the presence of 25‐hydroxyvitamin D_2_ in the CDC panel only; data on the presence of 25‐hydroxyvitamin D_2_ in samples assessed for serum vs. plasma comparison or in the precision panel are not available. While the aim of this study was to assess the technical performance of the Elecsys Vitamin D total III assay, future studies should consider the limitations mentioned here and investigate potential differences in the measurement of 25‐hydroxyvitamin D with respect to diverse patient demographics, and in samples from populations identified to be at risk of vitamin D deficiency.

## 5 CONCLUSION

The Elecsys Vitamin D total III assay demonstrated accuracy, good analytical performance in serum and plasma, and compared favorably with other commercially available assays, supporting its use as an aid for healthcare professionals in determining an individual's vitamin D status.

## AUTHOR CONTRIBUTIONS

Garnet Bending and Jill Grimme contributed to the study conception and design. Garnet Bendig, Jill Grimme, and Robert Christenson acquired the data. Peter Findeisen, Garnet Bendig, Jill Grimme, Elizabeth Moser, Christian Vogl, Robert Christenson, and Marta Leis contributed to the data analysis and/or interpretation. All authors provided a critical review of the study during development, accepted responsibility for the entire content of this study, and approved its submission.

## FUNDING INFORMATION

This study was funded by Roche Diagnostics International Ltd (Rotkreuz, Switzerland).

## CONFLICT OF INTEREST

Peter Findeisen and Marta Leis report no conflicts of interest. Garnet Bendig holds non‐voting equities in and is an employee of Roche Diagnostics GmbH. Christian Vogl holds non‐voting equities in and is an employee of Roche Diagnostics GmbH, and reports being an inventor of granted patents related to a method for the measurement of vitamin D and a release reagent for vitamin D compounds. Jill Grimme and Elizabeth Moser are employees of Roche Diagnostics. Robert Christenson received grants or contracts from Abbott Diagnostics, Beckman Coulter, Becton Dickinson Inc., Quidel Medical, Siemens Healthineers, and Sphingotech received consulting fees from Beckman Coulter, Becton Dickinson Inc., Quidel Medical, Roche Diagnostics, and Sphingotech; received payment or honoraria from Beckman Coulter, Becton Dickinson Inc., Quidel Medical, Roche Diagnostics, and Sphingotech; and held leadership or fiduciary roles in the American Association for Clinical Chemistry, the American College of Cardiology, and the Journal of Applied Laboratory Medicine.

## INFORMED CONSENT

Informed consent was obtained from all individuals included in the serum versus plasma sample analysis. For all other analyses, informed consent was not applicable.

## Supporting information


Table S1

## Data Availability

This study was conducted in accordance with applicable regulations. There may be ethical, legal, or other restrictions on sharing the de‐identified dataset used for our analysis. Please contact the corresponding author in case of queries.
