# Supplementary material for: A multicenter performance evaluation of the new Elecsys Vitamin D total III assay versus reference isotope dilution liquid chromatography tandem mass spectrometry and commercially available comparators
Source: J Clin Lab Anal. 2022 Jul 19;36(9):e24610. doi: 10.1002/jcla.24610 (PMC9459274; doi:10.1002/jcla.24610)
Supplement: Supplementary file 1 — Table S1 [file JCLA-36-e24610-s001.docx]

**SUPPLEMENTARY TABLE 1** Method comparisons between the Elecsys Vitamin D total III assay and comparator assays using the CDC ID-LC-MS/MS verification serum sample set

| **Comparator assay** | **n** | **Deming regression** | | **Pearson’s r** | **Bias at MDP of 30 ng/mL (%)** |
| --- | --- | --- | --- | --- | --- |
|  |  | **Intercept (95% CI)** | **Slope (95% CI)** |  |  |
| Access 25 (OH) Vitamin D Total assay | 116 | -1.92 (-4.07, 0.22) | 0.966 (0.89, 1.04) | 0.969 | -9.77 |
| ADVIA Centaur Vitamin D Total assay | 117 | -2.57 (-4.95, -0.18) | 0.963 (0.89, 1.04) | 0.958 | -12.3 |
| ARCHITECT 25-OH Vitamin D assay | 117 | 0.907 (-0.46, 2.28) | 0.921 (0.88, 0.97) | 0.982 | -4.88 |
| LIAISON 25 OH Vitamin D TOTAL assay | 117 | -5.26 (-7.87, -2.65) | 1.15 (1.06, 1.24) | 0.963 | -2.60 |

CDC, Centers for Disease Control and Prevention; CI, confidence interval; ID-LC-MS/MS, isotope dilution liquid chromatography tandem mass spectrometry; MDP, medical decision point.
